# The association between daily physical exercise and pain among women with fibromyalgia: the moderating role of pain catastrophizing

**DOI:** 10.1097/PR9.0000000000000832

**Published:** 2020-07-27

**Authors:** Asimina Lazaridou, Myrella Paschali, Kristin Schreiber, Laura Galenkamp, Michael Berry, Theodoros Paschalis, Vitaly Napadow, Robert R. Edwards

**Affiliations:** a Department of Anesthesiology, Harvard Medical School, Brigham and Women's Hospital, Chestnut Hill, MA, USA; b Department of Radiology, MGH/MIT/HMS Athinoula A. Martinos Center for Biomedical Imaging, Charlestown, MA, USA; c Department of Clinical Medicine, University of Cambridge, School of Clinical Medicine, Cambridge, United Kingdom

**Keywords:** Fibromyalgia, Psychological factors, Pain intensity, Physical exercise, Pain catastrophizing

## Abstract

**Introduction:**

Fibromyalgia (FM) is a condition marked by widespread chronic pain and an array of somatic and psychological symptoms. The primary objective of this study was to explore daily associations between physical activity and pain intensity among a sample of women with FM and the potential moderation of this association by pain catastrophizing.

**Methods:**

Women with FM (N = 107) completed questionnaires assessing pain, FM symptoms, and psychological measures and were then asked to report their levels of daily pain catastrophizing, physical activity, and pain intensity once per day for a period of 1 week using daily electronic diary-based tracking. In addition, objective measures of physical activity were collected using an activity tracker (Fitbit Flex), which measured step counts. Daily self-report physical activity was used as the independent variable and pain intensity (Brief Pain Inventory) was the outcome, whereas daily pain catastrophizing was tested in the model as the potential moderator.

**Results:**

Moderation analyses demonstrated associations between physical activity and pain intensity, which were moderated by patient's level of catastrophizing (B = 0.003, SE = 0.001, *P* < 0.05), with patients scoring higher in daily catastrophizing showing a relatively stronger link between higher day-to-day physical activity and increased daily FM pain. Significant associations were observed between pain catastrophizing, pain intensity, and Fitbit Flex step count (*P* < 0.05).

**Conclusions:**

Our findings suggest that increases in daily physical activity is associated with more self-reported pain intensity in women with FM pain, particularly among those with higher levels of pain catastrophizing.

## Abstract 2

Daily physical exercise is associated with more self-reported pain intensity in women with fibromyalgia pain, particularly among those with higher levels of pain catastrophizing.

## 1. Background

Fibromyalgia (FM) is a multifaceted, multifactorial chronic pain disorder characterized by various symptoms such as widespread pain, difficulty with sleep, cognitive difficulties, depression, anxiety, and fatigue.^18^ Fibromyalgia affects predominantly women, and the broad array of FM symptoms and contributory factors suggests a strong biopsychosocial basis for the condition. Many of the most effective treatments for FM pain symptomatology involve mind–body treatments that emphasize physical activity along with a cognitive and emotional focus (eg, yoga and movement therapies). Previous research on the role of exercise in FM has shown that short-term aerobic training can lead to improvements in physical function, and strength training can improve pain, wellbeing, and physical function, as well as decrease tender points and depression.^5^ Hence, current guidelines for patients with FM recommend individually tailored exercise programs including aerobic exercise and strength training.^8^ Generally, activity studies have demonstrated that patients with FM are physically deconditioned, with low levels of cardiorespiratory endurance and decreased muscle strength.^21^ Although activity has been identified as an important intervention for FM symptom management, patient adherence with recommended activity regimens are often suboptimal.

One model that can explain unhealthy behavior engagement in FM patients is the fear-avoidance model. The fear-avoidance model of chronic pain, introduced^39^ and further refined by Vlaeyen and colleagues,^53–55^ presents putative pathways by which patients who experience pain might become ensnared in a downward spiral of increasing avoidance, physical disability, and pain. It postulates that when bodily sensations, including movement, are misinterpreted in a catastrophic way (eg, pain reliably signals the presence of danger), pain-related fear increases, which is followed by the initiation of a number of safety behaviors including avoidance and guarded movements.^53^ In addition, avoidance behavior may hinder recovery because it diminishes the number of opportunities to correct patient's beliefs and expectations about pain, thereby allowing for wrongful anticipation of pain to persist.^13,36,38^ These interrelationships between fear-avoidance-related factors (such as catastrophizing) and outcome variables (such as physical disability) have been examined predominantly in cross-sectional studies using moderation and meditation analyses, and reviews have frequently called for further prospective research on these associations.^56,57^ Numerous past studies have explored pain catastrophizing as a moderator between pain-related clinical outcomes.^7,28,41,52^ Therefore, pain catastrophizing has since become increasingly recognized as an important moderator and one of the main determinants of the pain experience.

De Gier et al.^16^ demonstrated that in FM patients, pain-related fear is associated with decreased tolerance for physical performance, which in turn might explain avoidance behaviors of rigorous physical activities in these patients. Furthermore, FM patients seemed to be especially predisposed to psychological distress and present a unique tendency to pain catastrophize due to difficulty adjusting to the illness.^22^

There have not been many reports in the past that have examined the relationship between negative cognitions (eg, catastrophizing), engagement in physical activity, and self-reported pain especially in a sample of women with FM. The aim of the present daily diary study was to examine the associations among daily pain symptoms, catastrophizing, and physical activity in patients with FM. We hypothesized that catastrophizing would have important moderating effects on associations between physical activity and pain intensity in patients with FM.

## 2. Methods

In total, we invited 140 FM patients for telephone-based or in-person screening. Patients were diagnosed as having FM (as confirmed by physician and medical records) and met the 2011 American College of Rheumatology criteria, which require the presence of widespread pain as well as a number of other somatic (eg, sleep disturbance, fatigue), psychological (eg, depression, anxiety), and cognitive symptoms.^58^

After screening the 140 FM patients, 107 participants met the inclusion and exclusion criteria described below. This study was approved by the Partners Human Research Committee, and written informed consent was obtained from all participants. The inclusion criteria were as follows: (1) 18 to 75 years old, (2) female, (3) meet Wolfe et al.'s^58^ FM criteria for at least 1 year, (4) pain severity of at 3 out of 10 on average, (5) English language proficiency, and (6) able to provide written informed consent. The exclusion criteria included (1) comorbid acute pain or comorbid chronic pain condition more prominent than FM, (2) current use of stimulant medications, (3) pregnant or planning to become pregnant, (4) severe psychiatric disorder or prior psychiatric hospitalization in the past 6 months, (5) current or recent substance use disorder, (6) active suicidal ideation, and (7) recent lower-limb vascular surgery (this criterion was included as such surgery is a contraindication for some of the lower-body sensory testing procedures that were included—these findings are reported elsewhere).

### 2.1. Procedures

The baseline visit included engagement in the process of informed consent, completion of self-report questionnaires, and confirmation of study eligibility (described below). Sociodemographic information included date of birth, marital status, educational status, current occupational status, duration of pain symptoms, and medical comorbidities (Table 1). In addition, participants were asked to complete daily diaries for 7 days; responses to a variety of questions were recorded using a Likert scale. The Research Electronic Data Capture (REDCap) was used to assess daily pain and negative cognitions (eg, catastrophizing). Four surveys were used to assess key study variables: (1) Revised Fibromyalgia Impact Questionnaire (FIQR), (2) The Brief Pain Inventory (BPI), (3) Pain Catastrophizing Scale (PCS), and (4) Patient-Reported Outcomes Measurement Information System (PROMIS)-anxiety, PROMIS-depression.

**Table 1:**
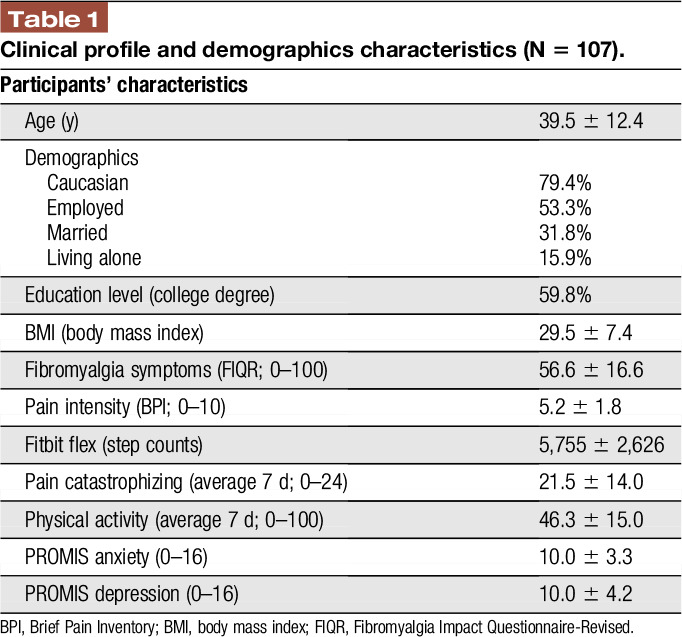
Clinical profile and demographics characteristics (N = 107).

#### 2.1.1. Fibromyalgia pain and symptoms

To measure FM symptomatology, including pain, we used the FIQR^3^ and the BPI.^12^ The FIQR is a 21-question assessment with an 11-point numeric rating scale of 0 to 10, with 10 being “worst.” The FIQR has 3 subscales for scoring: (1) “function”, (2) “overall impact,” and (3) “symptoms,” as well as a total overall score to assess the total impact of FM disease on the patient. The BPI is a 15-item measure, which includes 2 subscales that assess: (1) pain intensity and (2) pain interference^12^; the BPI is well validated in chronic pain.^48^

#### 2.1.2. Emotional distress—anxiety and depression

Participants completed the PROMIS anxiety and depression short forms, which have been repeatedly validated in chronic pain populations.^10,40^ The anxiety subscale consists of 7 items and assesses the frequency with which patients experience emotions such as fear, stress, and anxiety (“never” to “always”). The depression subscale consists of 8 items that assess the frequency with which the respondent has experienced emotions such as worthlessness, hopelessness, and sadness. Higher scores indicate more severe symptoms of emotional distress.

#### 2.1.3. Daily state pain catastrophizing

Participants were asked to report catastrophizing scores (0 “not at all” to 4 “all the time”; same response scale as used in the PCS) once a day for one week using a Likert scale through the secure REDCap. The Situational Catastrophizing Questionnaire (SCQ) is a six-question adaptation of the PCS (described above). It has been used by our group in a number of previous studies.^6,19^ The average of state pain catastrophizing across days was calculated.

#### 2.1.4. Daily physical activity

Patients were asked to report their levels of physical activity (0 “not at all” to 100 “all the time”) once a day for a period of 7 days using a Likert scale^27^ through the REDCap.

#### 2.1.5. Daily Fitbit Flex step counts (physical activity)

The Fitbit Flex is a popular, relatively inexpensive, and widely available small wristband that tracks physical activity, including measures of the estimated number of steps per day. The Fitbit Flex provides a valid measure of physical activity.^14,46,51^ The Fitbit Flex wirelessly synchronizes data to a computer, tablet, or phone and provides participants with feedback through a user-friendly website. Patients in the study wore the Fitbit Flex for the week during which they completed daily diaries; Fitbit Flex data were downloaded and stored at the end of the week. Participants optionally kept their Fitbit Flex after completion of the study. These data were used as part of an exploratory analysis to explore whether objective and subjective measures of physical activity are correlated.

#### 2.1.6. Body mass index

The body mass index (BMI) was calculated according to the following formula: BMI = body mass [kg]/(height [m])^2^.

### 2.2. Data analysis

All analyses were conducted using IBM-SPSS v.24. Descriptive data for continuous variables are presented as mean values and SDs, and data for categorical variables are presented as percentages (Table 1). Statistical assumptions and Pearson correlation coefficients were analyzed using SPSS (v. 23; IBM Corporation, Armonk NY) for MacVR. We conducted both bivariate correlations, controlling for age and education level, and the significance of associations did not differ. Thus, we only report bivariate correlations. To test our moderation model, we used the SPSS PROCESS macro because it allows to test moderation effects.^24^ Based on prior reports,^11,17,30,42,45,59^ we followed the approach of averaging the diary assessments across the 7 time-points. We tested Model 1, in which average daily physical activity was the predictor as used on past reports,^20,37^ pain intensity (BPI) was the dependent variable, and average daily state pain catastrophizing was the moderator of the relationship between average physical activity and average pain intensity controlling (covariates) for FM symptoms (FIQR) and BMI following prior literature.^25,50^

## 3. Results

Descriptive statistics for our study outcomes are presented in Table 1. The average age of patients was 39.5 years (SD = 12.4). Missing data percentages were low, with baseline questionnaires missing 1.7% and diary outcomes only 17.6% of the data. Table 2 demonstrated Pearson correlations for study outcomes and Table 1 demonstrates the mean values and SDs of the variables used in the moderation analysis described below. Fitbit data were collected from only 44 of 107 participants due to lack of compliance.

**Table 2:**
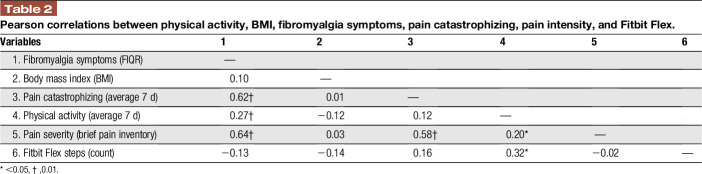
Pearson correlations between physical activity, BMI, fibromyalgia symptoms, pain catastrophizing, pain intensity, and Fitbit Flex.

### 3.1. Moderation analysis

**Figure 1.**
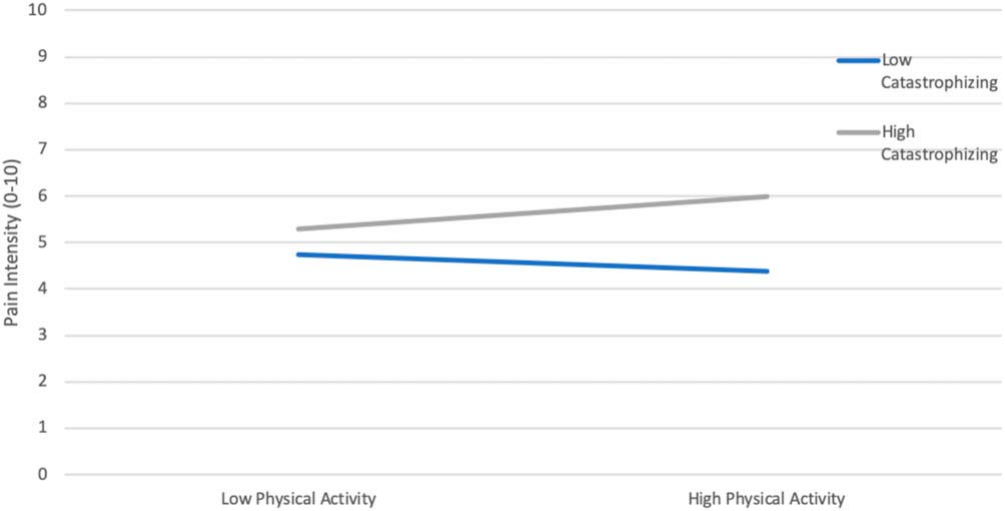
The significant effects of the interaction between daily pain physical activity and daily catastrophizing on pain intensity.

We examined whether state pain catastrophizing moderates the relationship between physical activity and pain intensity. Results showed that the overall model was significant (F (5, 87) = 17.81, *P* < 0.001, R^2^ = 0.50). Although the main effects of physical activity and pain catastrophizing were not significant (*P* > 0.05), the association between physical activity and pain intensity was moderated by state pain catastrophizing. To further examine the significance of simple slopes of the interaction, a simple moderation model was first conducted, and results showed the interaction was significant (B = 0.003, SE = 0.001, *P* < 0.05). These results suggest that the association between physical activity and pain intensity is moderated by state pain catastrophizing (Table 3). A visual representation of the moderation is presented in Figure 1, depicting the association between physical activity and pain as a function of state pain catastrophizing (ie, 1 SD above and below the mean; Fig. 1). In addition, significant associations were observed between average state pain catastrophizing, pain intensity, and Fitbit Flex step count (*P* < 0.05). Self-report physical activity was positively correlated with average Fitbit Flex step count (*P* < 0.005).

**Table 3:**
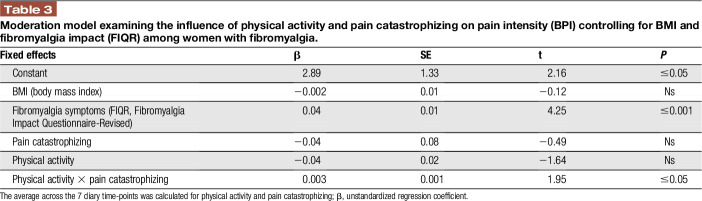
Moderation model examining the influence of physical activity and pain catastrophizing on pain intensity (BPI) controlling for BMI and fibromyalgia impact (FIQR) among women with fibromyalgia.

## 4. Discussion

The aim of this study was to explore the moderating effects of catastrophizing on the relationship between daily physical activity and pain intensity in patients with FM. The primary finding of our diary-based study was that catastrophizing moderated the relationship between daily self-reported physical activity and pain intensity. State pain catastrophizing and physical activity both seem to influence pain levels in FM, with catastrophizing magnifying the acute, pain-increasing effects of physical activity. Patients high in state pain catastrophizing report greater pain in the context of relatively higher activity levels, whereas among patients low in state pain catastrophizing, higher activity levels are associated with lower ratings of pain intensity. Our results support previous findings about the importance of psychosocial factors including anxiety, depression, and catastrophizing that should be targeted in chronic pain patients to pain-related disability and sedentary behaviors.^27,34^

A primary challenge to effective self-management of chronic pain is limited adherence to physical activity and lifestyles that are most likely to reduce the physical and emotional “triggers” that worsen symptoms in chronic pain.^2,9,44^ We now have abundant evidence that negative affective and cognitive states contribute to reduced activity levels in patients with chronic pain.^15^ Although we do not have data on the mechanisms by which these associations unfold, it is possible that high catastrophizing may magnify the perception of pain when physical activity levels are higher, promoting a feeling of reduced motivation and increased physical symptoms. Past evidence has indicated that high pain catastrophizing predicts deficits in activity-associated hypoalgesia,^4^ which may contribute to our observations of larger activity-provoked increases in pain among patients with high catastrophizing.

Furthermore, because fear of movement due to potential injury represents a response to pain related to state catastrophizing,^54^ this could be a further mechanism through which state catastrophizing can increase perceived pain intensity when there is increased physical activity. Pain perception in individuals with higher pain-related fear and higher self-reported state catastrophizing seems to be biased towards predictions (eg, cognitive bias) and less towards nociceptive inputs.^32^ Therefore, on higher activity days, higher state catastrophizing is likely to produce increases in perceived pain. Catastrophizing has also been shown to be significantly associated with fatigue and to serve as a good predictor of fatigue severity,^33^ which may be one of the pathways by which state catastrophizing reduces engagement in daily activity. Finally, pain catastrophizing seems to have a maladaptive influence on brain circuitry that is involved with pain facilitation and central sensitization.^26,29^ Therefore, it seems that it is important to assess catastrophizing and target it when focusing on improving physical function in patients with FM.^35,47,49^

In addition, it is important to note that measuring physical activity is inherently challenging, regardless of the method of measurement. Evaluating patient-reported activity estimates and accelerometer-monitored steps seems to yield overlapping yet substantially distinct domains of information. It is interesting to note that patient-reported daily activity is correlated with pain intensity and with other FM symptoms as well as Fitbit-assessed step count. Although the subjective and objective measures of physical activity were modestly and significantly correlated in this study, they seem to have different patterns of association with other subjective reports of FM symptomatology. Although some have argued that accelerometry may offer a better assessment of physical activity than subjective self-report because it avoids issues of recall bias, there are notable limitations for objective physical activity assessments, including the inability to assess dynamic physical activity, dependence on accelerometer wear location (eg, wrist vs hip), and sensitivity to the assessment variables analyzed (eg, step count, activity counts).^31^ In addition, repeated assessment of repeated physical activity and situational catastrophizing from a multilevel modelling analysis approach would have been able to capture day-to-day variability among participants suffering from FM.

Collectively, the limitations of this study should be recognized when interpreting these results. First, physical activity over a 7-day period may not be fully representative of habitual physical activity levels. Second, participants used in our study included only women with moderate to severe FM pain; this sample may not fully represent all FM populations, such as those with milder FM symptoms or men. Finally, this study is based on baseline data assessing patients' daily symptoms. This relatively brief assessment of FM symptomatology is likely unable to capture the extensive range of FM outcomes (and the mechanisms that contribute to those outcomes) that emerge over longer timeframes. In addition, the numeric scale of physical activity assessment used in our study has not been validated before as an objective measure.

## 5. Conclusions

Overall, given the physical limitations imposed by pain, catastrophizing seems to be an important outcome to target with chronic pain interventions because there are strong associations with cardiovascular and respiratory conditions.^43^ Aerobic activity, stretching, strengthening, and aqua therapy have been shown to reduce pain and improve functional capacity and quality of life.^1,23^ Future interventions combining skills targeting physical activity and reducing negative maladaptive pain cognitions (eg, Cognitive Behavioral Therapy) might be able to reduce kinesiophobia and promote a healthier lifestyle in women with chronically painful musculoskeletal conditions such as FM.

## Disclosures

The authors have no conflicts of interest to declare.
